# A non-redundant data set of nanobody-antigen crystal structures

**DOI:** 10.1016/j.dib.2019.103754

**Published:** 2019-03-06

**Authors:** Uroš Zavrtanik, San Hadži

**Affiliations:** Department of Physical Chemistry, Faculty of Chemistry and Chemical Technology, University of Ljubljana, 1000 Ljubljana, Slovenia

## Abstract

A non-redundant data set of nanobody-antigen crystal structures is presented. The data set consists of a collection of cleaned pdb files which can be readily used as an input with most automatic analysis software. The accompanying data also include nanobody amino acid sequences with the annotated CDR regions. In the tabular format, we provide data on the interaction properties for each complex such as number of intermolecular interactions, experimental affinity and changes of the solvent accessible area. We also include the data regarding the surface composition of all nanobody and antigen molecules (surface occurrence of each amino acid type and its secondary structure). The data may be used for further structural bioinformatic studies of nanobodies and as the reference data when performing comparisons with the conventional antibodies.

**Table:** Specifications table

Subject area	*Biochemistry*
More specific subject area	*Structural bioinformatics, Immunoinformatics*
Type of data	*A collection of atom coordinates in the pdb format, tables, text files*
How data was acquired	*Survey of the nanobody-antigen crystal structures deposited in the Protein Data Bank*
Data format	*Raw and analyzed*
Experimental factors	*A Protein Data Bank survey was performed in January 2018. The non-redundant data set consists of 123 structures in the pdb format. The atom coordinate files were cleaned as described below and as such may be used for further large-scale automatic analysis.*
Experimental features	*Nanobody CDR regions were determined using program ANARCI. Molecular surface calculations were performed using program NACCESS with the default parameters. Intermolecular contacts were found using the Biopython modules. Secondary structure of residues was assigned using DSSP as incorporated in the Biopython module “Biopdb.DSSP”.*
Data source location	*University of Ljubljana, Ljubljana, Slovenia*
Data accessibility	*Data is given in this paper.*
Related research article	*U. Zavrtanik, J. Lukan, R. Loris, J. Lah, S. Hadži, Structural Basis of Epitope Recognition by Heavy-Chain Camelid Antibodies, J. Mol. Biol. 430 (2018) 4369–4386. doi:**10.1016/J.JMB.2018.09.002**.*

**Table:** 

**Value of the data** • A non-redundant dataset of 123 nanobody-antigen crystal structures is presented. • The atomic coordinate files in the pdb format are processed in a way which is suitable for most analysis programs. • Nanobody amino acid sequences and the sequences of CDR regions are provided. • Accompanying data for each complex provide the information on the interaction properties including the experimentally determined affinities obtained from the literature.

## 1 Data

Heavy-chain antibody fragments from camelids, also known as nanobodies, are much smaller (15 kDa) than conventional antibodies and due to their small size have found numerous applications, particularly as crystallization chaperones [1], [2]. We recently analyzed various structural properties of the nanobody-antigen complexes based on the non-redundant data set of the crystal structures [3]. This data set is presented in present report.

The Data_set.zip file contains a collection of cleaned pdb files of the nanobody-antigen complex with each filename starting with the original pdb code (xxxx_3.pdb). The original dataset consisted of 105 nanobody-antigen structures retrieved in early 2018 from Protein Data Bank. Since our first search 18 new complexes have been deposited to PDB, which are now provided in addition under the folder 'extended dataset'. Beside the coordinate file of the nanobody-antigen complex we also include atomic coordinates of the separated molecules (nanobody and antigen) with the xxxx_1.pdb and xxxx_2.pdb filenames for the antigen and nanobody, respectively. Note that the structure 4grw.pdb contains three nanobodies bound to different binding sites of the antigen. This structure was therefore split into three structures, each corresponding to one binding interface.

All nanobody sequences from the data set are provided in the Nb_seq.fasta file in the FASTA format. Each sequence in named according to the corresponding pdb file (xxxx_2pdb). CDR sequences (CDR 1, CDR 2 and CDR 3) of nanobodies are provided in the separate file (CDR_sequences.txt).

Additional information related to the data set is summarized in three tables in the .xlsx format. Table 1 contains the relevant information regarding the interaction properties. In the columns, the following information is provided: pdb code, total number of intermolecular contacts, number of intermolecular contacts involving residues from the CDR 1, 2, 3 and those residues which are not part of CDR (non-CDR). The following columns list the change in the solvent accessible surface area (SASA) upon complex dissociation, change in SASA of the main chain atoms, change in SASA of the side chain atoms, total change in the polar and nonpolar SASA. The last columns in Table 1 report on the free energy (Δ*G*) of complex dissociation at 25 °C, for cases where literature values were available. Additionally, in the cases where enthalpy and entropy contributions were reported these are also included.

Tables 2a(nb).xlsx in 2b(ag).xlsx provide the information related to the surface composition of the nanobodies and antigens, respectively. For each complex, the protein surface is divided into two categories: the whole surface and the contact surface (termed epitope for antigen and paratope for nanobody). The contact surface consists only of the surface residues mediating the intermolecular contacts. For each surface category (whole or contact) the columns list occurrence of each amino acid type as the well as number of residues with particular secondary structure conformation as classified by DSSP.

## 2 Experimental design, materials and methods

### 2.1 Generation of the nanobody-antigen data set

The survey of the nanobody-antigen structures was made on the global repository of PDB (www.rcsb.org) in January 2018 and was further updated in December 2018 using search words: *nanobody*, *camelid heavy-chain antibody* and *single-domain antibody*. Following filters were further applied in the PDB search: Experimental method = X-ray, X-ray Resolution = 0–3 Å and Stoichiometry = heteromer. These searches resulted in 217 hits which were further filtered. First, we deleted all complexes with >90% identity score of Nb sequences (using computer program CD-HIT [4]), to obtain only the unique binding surfaces. Second, all structures were also checked manually, to ensure that we retrieved only the complexes with the relevant biological interfaces and to avoid analysis of the crystal contacts.

### 2.2 Processing of the pdb files

The non-redundant data set consists of 123 nanobody-antigen crystal structures with atomic coordinate files in the pdb format. The original pdb files (as retrieved from the data bank) often contain some extraneous information which leads to the errors when analyzing the data using most programs and scripts. Therefore, here we provide the cleaned pdb files, which were processed as follows:

1) when multiple complexes were present in the asymmetric unit only the first listed complex was retained,
2) all information (HEADER, TITLE etc) in the pdb files except the ATOM records were removed,
3) all hydrogen atoms were removed,
4) water molecules, ligands and other compounds (designated as HETATM records) were removed,
5) residues with the alternative conformations and those with zero occupancy were removed.

Chain and atom numbering was retained as in the original pdb file, so that the molecular structures in the processed files can be traced back to the original file.

### 2.3 Assignation of CDR regions

CDR regions (CDR 1, CDR 2 and CDR 3) of nanobodies were determined using standard IMGT numbering as implemented in the ANARCI computer program [5], [6].

### 2.4 Changes in solvent accessible surface and intermolecular contacts

All surface calculations were preformed using NACCESS version 2.1.1 using the default parameters [7]. Calculations of the SASA were done using the whole complex (xxxx_3.pdb files) and using the separated molecules (xxxx_1.pdb and xxxx_2.pdb files). Changes in the SASA were calculated as a sum of SASA of the molecules in the separated form minus the SASA of the complex. Nanobody and antigen surface residues are defined as those where the residue exposure is above 50 Å^2^ (for the molecules in the separated form). Contacting residues are those which are both solvent exposed (SASA>50 Å) in the isolated form and have one of its atoms located less or equal to 5 Å away from any atom in the partner molecule (Nb or Ag) in the complex. Nanobody residues involved in the intermolecular contacts constitute the paratope surface while those from antigen the epitope surface.
